# A Novel Amperometric Glutamate Biosensor Based on Glutamate Oxidase Adsorbed on Silicalite

**DOI:** 10.1186/s11671-017-2026-8

**Published:** 2017-04-07

**Authors:** O. V. Soldatkina, O. O. Soldatkin, B. Ozansoy Kasap, D. Yu. Kucherenko, I. S. Kucherenko, B. Akata Kurc, S. V. Dzyadevych

**Affiliations:** 1 Institute of High Technologies, Taras Shevchenko National University of Kyiv, Volodymyrska Street 64, Kyiv, 01003 Ukraine; 2 Institute of Molecular Biology and Genetics of NAS of Ukraine, Zabolotnogo Street 150, Kyiv, 03143 Ukraine; 3 Micro and Nanotechnology Department, Middle East Technical University, 06531 Ankara, Turkey; 4 Central Laboratory, Middle East Technical University, 06531 Ankara, Turkey

**Keywords:** Silicalite, Enzyme, Biosensor, Glutamate oxidase, Amperometry

## Abstract

In this work, we developed a new amperometric biosensor for glutamate detection using a typical method of glutamate oxidase (GlOx) immobilization via adsorption on silicalite particles. The disc platinum electrode (*d* = 0.4 mm) was used as the amperometric sensor. The procedure of biosensor preparation was optimized. The main parameters of modifying amperometric transducers with a silicalite layer were determined along with the procedure of GlOx adsorption on this layer. The biosensors based on GlOx adsorbed on silicalite demonstrated high sensitivity to glutamate. The linear range of detection was from 2.5 to 450 μM, and the limit of glutamate detection was 1 μM. It was shown that the proposed biosensors were characterized by good response reproducibility during hours of continuous work and operational stability for several days. The developed biosensors could be applied for determination of glutamate in real samples.

## Background

Glutamate (glutamic acid) plays an important role in vital activity of humans and other mammals, especially in the functioning of the central nervous system. In particular, glutamate is the major excitatory neurotransmitter in the central nervous system of mammals. It also has a significant effect on nitrogen metabolism. The concentration of glutamate in certain parts of the body may influence the development of heart attacks, strokes, and various neuropathological states [1, 2].

Glutamate is part of many pharmaceuticals due to its ability to sensitize the taste receptors and stimulate the brain activity. A lot of foodstuff contains small amounts of glutamate [3, 4], which gives food “beef” taste. Therefore, glutamate is often used as a flavor enhancer. This is why it is rather problematic to completely eliminate glutamate from the diet. In glutamate-sensitive people, the so-called “Chinese restaurant syndrome” may develop [5, 6]. Glutamate badly affects the retina and can contribute to vision loss.

Determination of glutamate is of significance in clinical biochemistry when diagnosing the diseases associated with abrupt changes of glutamate level in the body, including diseases of liver and cardiovascular system [5, 7]. In clinical laboratories, glutamate is used to determine the activity of some aminotransferases.

The scope of practical application of glutamate is continuously growing. The methods of accurate and rapid detection of glutamate are required in neurophysiology and neuropathology, fundamental and clinical medicine, pharmaceutical and food industries, and in analytical biochemistry and biotechnology [1, 5, 7].

The up-to-date standard methods for highly accurate determination of glutamate, spectrophotometry and liquid chromatography, require qualified personnel and complex expensive equipment [1, 5, 8, 9].

Additionally, chemiluminiscence can be also used, which includes the application of luminol, potassium ferricyanide, and luminophotometer [10]. Oxygen consumption at the glutamate oxidation can be fixed with an oxygen fiber optic sensor, which registers the changes in luminescence of a deposited layer sensitive to the oxygen concentration [3]. The method used for glutamate determination in meat and meat products is based on two enzymatic reactions resulting in the glutamate oxidation and formation of a colored compound formazan, the concentration of which is measured with a spectrophotometer.

The disadvantage of the above methods is the requirement of rather difficult pretreatment of analyzed samples and their unsuitability for rapid analysis of large amount of samples and for real-time monitoring. New bioanalytical devices, biosensors, can be considered as a promising alternative to the methods mentioned [11].

Among electrochemical biosensors, the amperometric ones are considered to be the most promising, and they are most often used to determine glutamate [1, 4, 5, 7, 12]. Besides, the potentiometric electrodes (NH^4+^ detection) can be an alternative; however, they are less sensitive. For selective determination of glutamate in the brain, it was developed the system of platinum microelectrodes covered with electropolymerised hyperoxidated polypyrrole, which were immobilized on ceramics [13]. An automatic flow-injection system in biosensor devices can be useful in the monitoring of glutamate determination in real-time [5, 14]. The multi-channel biosensor system was created for dynamic identification of several components (including glutamate) in food production. Modified graphite electrodes with stabilizing additives were used to provide stable function of the biosensor at long-term storage [15]. In most studies on the development of glutamate biosensors, the enzyme L-glutamate oxidase of different origin is used [16–18]. The enzymes glutamate decarboxylase, glutamate dehydrogenase, and glutamate synthetase are also used [1], but glutamate oxidase far exceeds them in characteristics.

Currently, a number of biosensors and biosensor systems have been developed for glutamate determination in various real samples—foods and pharmaceuticals [1, 7], cell cultures [12, 19], blood serum and urine [1, 5], microdialyzates at neurophysiological studies [2, 20], and for monitoring fermentation in the food industry [21, 22]. However, many of these biosensors are based on a complex and time-consuming method of immobilization, often with the use of toxic reagents. Neither of them has not been commercialized so far. Therefore, the elaboration of new methods of creating glutamate-sensitive biosensors with improved analytical characteristics is an actual challenge.

This study is aimed at creation of the amperometric biosensor for glutamate determination, which allows faster and more accurate analysis and can be suitable for mass production in future. The problem is supposed to be solved using a new method of enzyme immobilization, the glutamate oxidase adsorption on transducers covered with a silicalite layer.

For the first time, the efficiency of various types of zeolites as carriers for the enzyme immobilization has been shown when developing conductometric biosensors [23–27]. The procedure of enzyme adsorption on silicalite was tested for a number of enzymes—acetylcholinesterase [27], urease [23, 25], recombinant urease [26], and butyrylcholinesterase [27]. Additionally, the effectiveness of this technique has been shown for enzyme biosensors based on pH-sensitive field-effect transistors [28–32]. Moreover, it was revealed that some zeolites can be useful for glucose oxidase adsorption in amperometric biosensors for glucose determination [33]. Therefore, an attempt was made to apply this method of immobilization for the development of amperometric glutamate-sensitive biosensor with improved analytical characteristics using GlOx adsorbed on silicalite.

## Methods

### Materials

Glutamate oxidase (GlOx, EC 1.4.3.11) from *Streptomyces sp*., activity 7 U/mg (Yamasa Corporation, Tokyo, Japan) was used in biorecognition elements of biosensors. Bovine serum albumin (BSA, fraction V), glycerol, ascorbic acid, HEPES, and 50% aqueous solution of glutaraldehyde (GA) have been received from Sigma-Aldrich Chemie (Germany). L-glutamate was from Affymetrix (USA). All other chemicals were of analytical purity grade.

### Synthesis of Silicalite Crystals

The molar composition of the clear solution used for synthesis of silicalite crystals is 1TPAOH: 4TEOS: 350H_2_O. Hydrolysing tetraethoxysilane (TEOS) with tetrapropylammonium hydroxide (TPAOH) at a constant stirring for 6 h at room temperature, we obtained a homogeneous solution. The solution was introduced into Teflon-lined autoclaves. The crystallization took place at 125 °C during 1 day. The material, which did not react, was removed from the solution by centrifugation. The size of silicalite particles was approximately 400 nm.

### Characterization of Silicalite

**Fig. 1:**
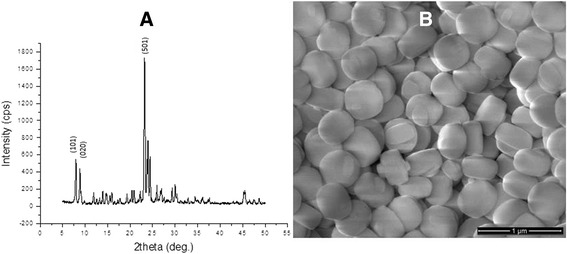
XRD spectrum (**a**) and SEM image (**b**) of synthesized silicalite

The resulting samples were characterized by powder X-ray diffraction (XRD) using Ni filtered Cu-Kα radiation in a Philips PW 1729 X-ray diffractometer. Scanning electron microscopy (SEM) analysis was performed in a 400 Quanta FEI after gold-palladium coating using Polaron Range Sputter Coater. The energy dispersive X-ray spectroscopy (EDX) analyses was carried out utilizing a Phoenix EDAX X-ray analyzer equipped with the Sapphire super ultrathin window detector attached to the Hitachi S-4700 FE-SEM. Dynamic light scattering (Malvern Mastersizer 2000) was used to determine the particle size distribution of silicalite. The nitrogen adsorption/desorption experiments were carried out using the NOVA 3000 series (Quantachrome Instruments) instrument. Surface area of silicalite was studied by Multipoint BET, whereas pore size and volume—the Saito-Foley (SF) and t-plot methods. Sample preparation included outgassing samples under vacuum at 300 K for 4 h before analysis. According to the X-ray diffraction data presented in Fig. 1a, silicalite exhibited characteristic diffraction lines of their structures. In Fig. 1b, the morphology of silicalite was observed to be more of a round plate shape of around 400 nm in diameter and 180 nm in thickness with clear surface. According to particle size distribution analysis, these particles were in the 150–1100 nm size range. In Table 1, Si/Al ratios, particle sizes, pore sizes, external and total surface areas, micro-, and mesopore volumes are given.

**Table 1:** Characteristics of silicalite

Sample name	Si/Al^a^	Part. size (nm)^b^	Pore size (nm)^c^	*S*_ext_ (m^2^/g)^d^	*S*_total_ (m^2^/g)^e^	Pore volume (cc/g)^c^
Silicalite	No Al	400^*^	0.45	52	185	0.08

^*^Average diameter of the particle

^a^Measured by EDX

^b^Measured by SEM

^c^Measured by Saito-Foley (SF) method

^d^Measured by t-plot method

^e^Measured by BET

### Design of Amperometric Transducers

**Fig. 2:**
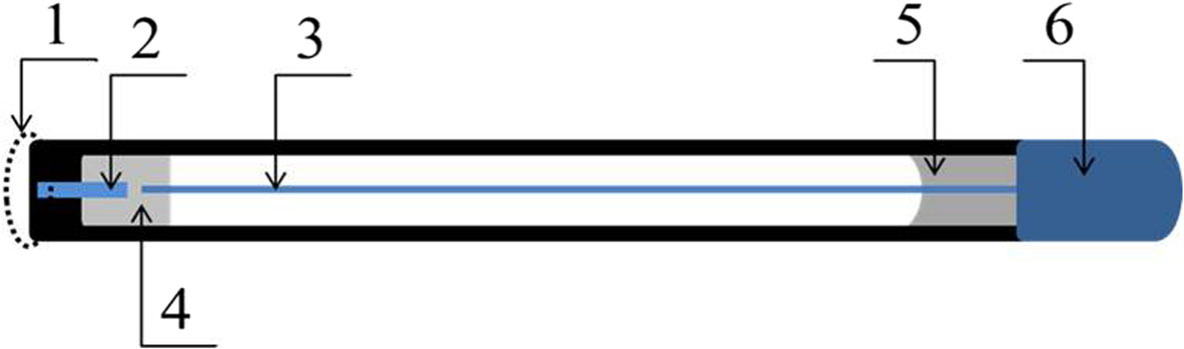
Scheme of platinum disc electrodes. **1** Bioselective membrane, **2** platinum wire, **3** inner conductor, **4** wood alloy, **5** epoxy resin, and **6** contact panel

In this work, the platinum disc electrodes of own production served as amperometric transducers (Fig. 2). A platinum wire of 0.4 mm in diameter and 3 mm long was sealed in the end of a glass capillary with an outer diameter of 3.5 mm. An open end of the wire served as the transducers working surface. An inner end of the platinum wire was connected to a copper wire, placed inside the capillary, using fusible Wood’s alloy. A contact pad for connecting the electrode to the measuring setup was placed at the other end of the copper wire. The working surface of platinum electrodes was cleaned by immersing it into a concentrated sulfuric acid for 30 s, washing with water and ethanol-wetted cotton swab prior to immobilization of the bioselective element. The surface of working electrode was obtained by grinding with alumina powder (particles of 0.1 and 0.05 μm) and treated with alcohol before the bioselective element immobilization. The electrode surface was periodically restored using the same grinding procedure.

### Drop-Coating Transducers with Silicalite

A silicalite layer was formed on the transducer surface by dip-coating. 2.5% silicalite suspension in 20 mM HEPES, pH 6.5, was used. 0.15 μl of the solution were deposited onto active zones of transducer, then it was heated during 1–1.5 min to 150 °C. This temperature had no effect on silicalite and did not influence the transducer working parameters.

### Enzyme Adsorption on Silicalite

0.1 μl of 4% GlOx solution in 20 mM HEPES, pH 6.5, were deposited onto the active zone of transducer previously coated with silicalite, then the transducer was exposed to complete air-drying (for 5 min). Neither glutaraldehyde nor any other auxiliary compounds were used. Next, the transducers were submerged into the working buffer for 5–10 min to wash off the unbound enzyme. After the experiments, the transducer surface was cleaned from silicalite and adsorbed enzyme with ethanol-wetted cotton.

### Experimental Setup for Amperometric Measurements

**Fig. 3:**
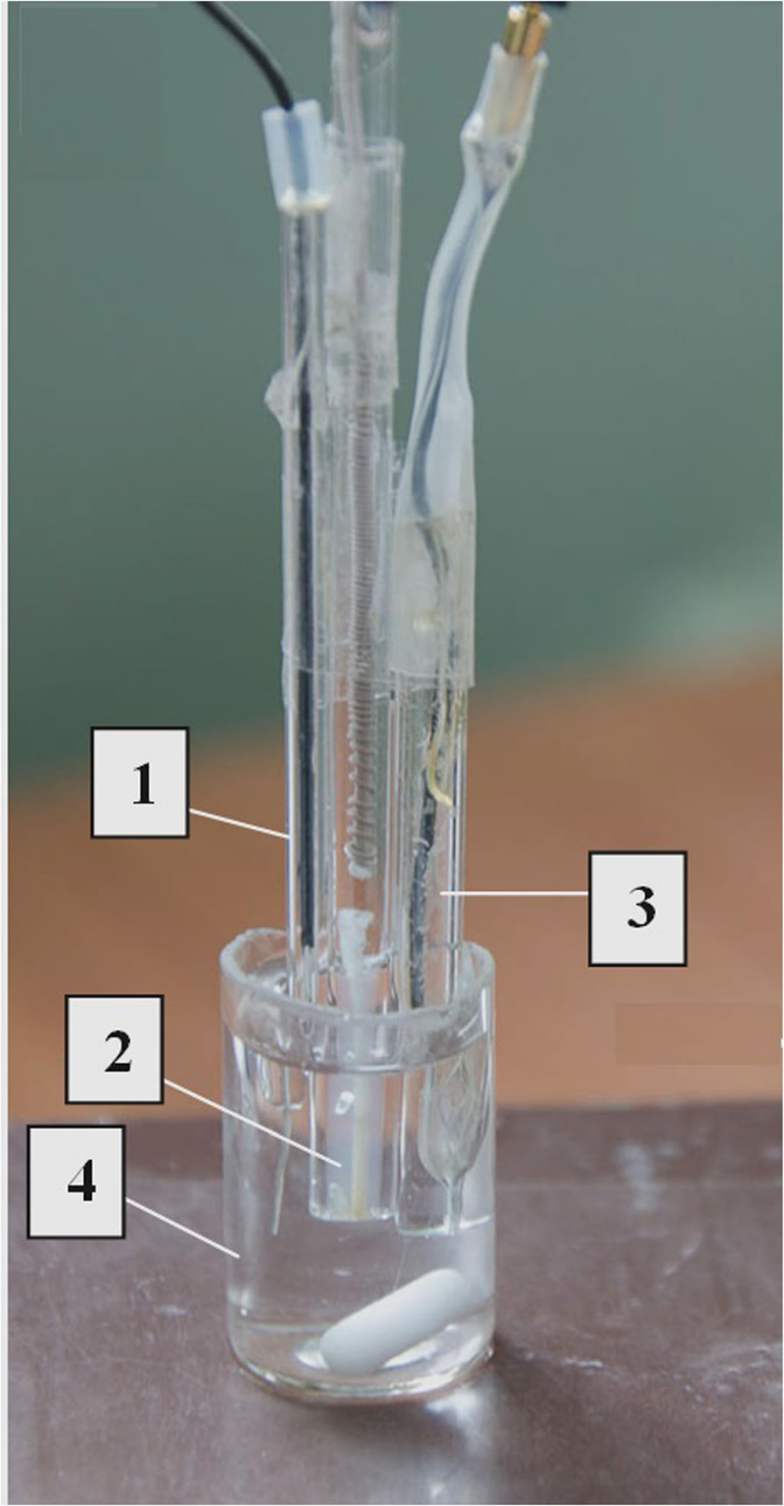
General view of measuring cell with three electrodes (**1** auxiliary electrode, **2** Ag/AgCl reference electrode, **3** working electrode, and **4** working cell with 2 ml of buffer solution and magnetic stirrer)

A three-electrode scheme of amperometric analysis was used. The developed working amperometric transducers were connected to the PalmSens potentiostat (Netherlands) along with the auxiliary platinum electrode (with a much larger area of the platinum surface compared to the working electrode) and the Ag/AgCl reference electrode (Fig.3).

### Measurement Procedure

Measurements were carried out in 20 mM HEPES, pH 7.4, in a chronoamperometric mode (“amperometric detection”) at a constant potential of +0.6 V vs Ag/AgCl reference electrode in an open cell with vigorous stirring. The substrate concentration in the measuring cell was specified by the introduction of aliquots of the substrate standard stock solution to the working buffer. All experiments were performed in at least three series.

## Results and Discussion

The operation of amperometric biosensor for glutamate determination is based on the enzymatic reaction (1) in a bioselective membrane, which consists in the glutamate oxidation and the formation of electrochemically active hydrogen peroxide. When applying positive potential, the reaction of hydrogen peroxide oxidatoion on the electrode (2) resulting in the generation of electrons, which are directly registered via amperometric transducer:

1 $$ \begin{array}{l}\mathrm{Glutamate}\ \mathrm{oxidase}\\ {}\mathrm{Glutamate} + {\mathrm{O}}_2\to \upalpha \hbox{-} \mathrm{ketoglutarate} + {\mathrm{NH}}_3 + {\mathrm{H}}_2{\mathrm{O}}_2\end{array} $$

2 $$ \begin{array}{l}+600\ \mathrm{mV}\\ {}{\mathrm{H}}_2{\mathrm{O}}_2\to 2{\mathrm{H}}^{+} + {\mathrm{O}}_2 + 2{\mathrm{e}}^{\hbox{-}}\end{array} $$

At the first stage of this work, the method of enzyme adsorption on silicalite was optimized for creating amperometric GlOx-based biosensor for glutamate determination. The enzyme amount adsorbed on a transducer depends in the first place on the amount of sorbent (silicalite). The size of silicalite layer is a function of both its concentration in solution and the time of layer formation.

**Fig. 4:**
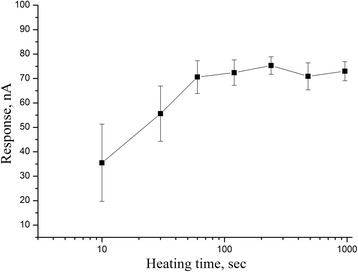
Dependence of responses of biosensor based on GlOx, adsorbed on silicalite, on the time of silicalite deposition on transducer. Measurements in 20 mM HEPES buffer, pH 7.4, at a constant potential of +0.6 V vs Ag/AgCl reference electrode. Glutamate concentration, 1 mM

First, the procedure of deposition of 5% of silicalite on the transducer was carried out with different time duration of heating at 150 °C from 10 to 960 s in the oven. It allowed a controllable increase in amount of silicalite on transducer and made it possible to explore an effect of this option on the final biosensor sensitivity to glutamate (1 mM). The dependence of biosensor responses on the time of silicalite heating on the electrode surface is shown in Fig. 4. As seen, the time of silicalite layer formation on the platinum electrode surface should be at least 60–90 s.

**Fig. 5:**
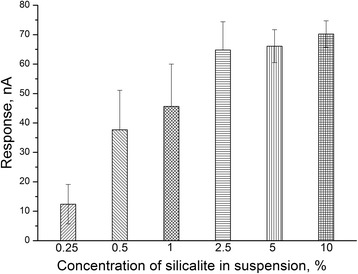
Dependence of responses of biosensor based on GlOx, adsorbed on silicalite, on concentration of silicalite deposited on transducer during 90 s. Measurements in 20 mM HEPES buffer, pH 7.4, at a constant potential of +0.6 V vs Ag/AgCl reference electrode. Glutamate concentration, 1 mM

Next, it was necessary to determine the optimal concentration of silicalite when forming its layer on the surface of platinum disc electrode of GlOx-based biosensor (Fig. 5). Deposition of 2.5% silicalite significantly increased the bioselective element activity compared with 0.25% silicalite, but higher silicalite concentration had no notable effect. Therefore, in further studies, GlOx was adsorbed on the amperometric transducers covered with 2.5% silicalite suspension.

The next task was to find the optimal conditions of GlOx adsorption on silicalite, i.e., the time of procedure and enzyme concentration. The adsorption efficiency was assessed by measuring the biosensor responses. Despite our assumption about significant dependence of the adsorption efficiency on the time, the value of biosensor responses was about the same at the adsorption time ranging from 2 to 30 min. Five minutes was taken as optimal value because it was enough for complete drying of the enzyme drop deposited on the transducer.

**Fig. 6:**
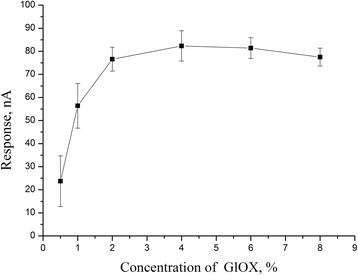
Dependence of responses of biosensor based on GlOx, adsorbed on silicalite, on enzyme concentration at membrane deposition. Measurements in 20 mM HEPES buffer, pH 7.4, at a constant potential of +0.6 V vs Ag/AgCl reference electrode. Glutamate concentration, 1 mM

An effect of the enzyme concentration was more remarkable. Figure 6 shows the experimental results regarding an influence of GlOx concentration during adsorption on the biosensor responses. As it can be seen, an increase of GlOx concentration in HEPES solution from 0.5 to 4% led to almost four-times increase in the biosensor response because of the increased amount of adsorbed GlOx. However, GlOx concentrations higher than 4% did not improve the results, probably because of an excess of the enzyme. Thus, 4% GlOx solution can be considered as optimal for the biosensor creation.

**Fig. 7:**
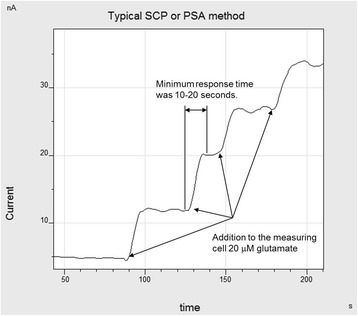
Typical responses of biosensor, based on GlOx adsorbed on silicalite, to 20 μM glutamate. Measurements in 20 mM HEPES buffer, pH 7.4, at a constant potential of +0.6 V vs Ag/AgCl reference electrode

After optimization of the procedure of coating of the amperometric sensor with silicalite layer and GlOx adsorption on silicalite, it was interesting to check main analytical characteristics of the obtained biosensor for glutamate determination. To demonstrate the biosensor work under optimal conditions, typical responses of the biosensor, to 20 μM glutamate is shown in Fig. 7. As seen, the measured responses were quite pronounced with a slight noise of baseline. In our view, the fluctuations of signal level after adding glutamate are because of either the noise caused by stirrer or the variations of oxygen level in the membrane. The minimum response time was 20–30 s.

**Fig. 8:**
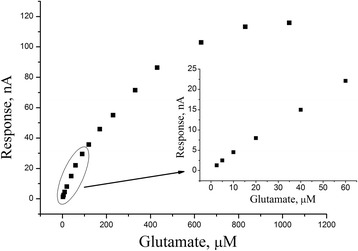
Calibration curve of biosensor, based on GlOx adsorbed on silicalite, for glutamate determination. Measurements in 20 mM HEPES buffer, pH 7.4, at a constant potential of +0.6 V vs Ag/AgCl reference electrode

The typical calibration curve of glutamate with the GlOx/silicalite biosensor is shown in Fig. 8. The linear concentration range was 2.5–400 μM. The biosensor sensitivity to glutamate was 0.5 nA/μM. The typical developed biosensor had the detection limit about 0.5–1 μM (it was measured as the glutamate concentration, the response to which is three times larger than the baseline noise).

**Fig. 9:**
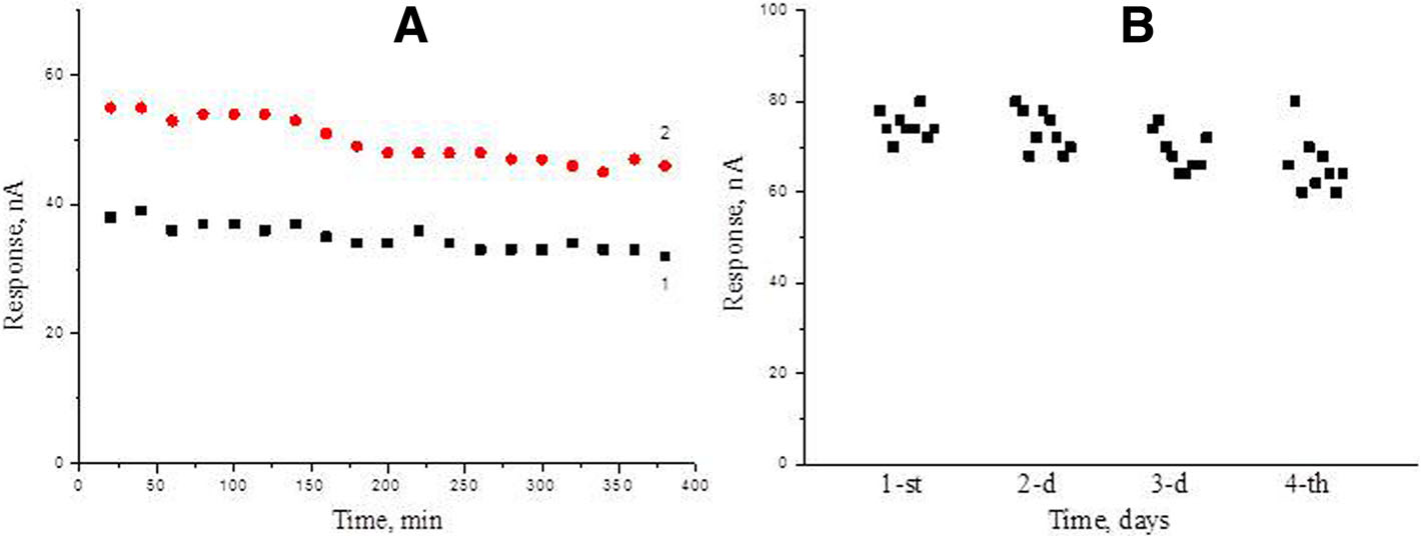
Reproducibility of biosensor responses to 0.5 mM (1) and 1 mM (2) glutamate during one working day (**a**) and operational stability of the biosensor responses to 1 mM glutamate during four days (**b**). Measurements in 25 mM HEPES buffer, pH 7.4, at a constant potential of +0.6 V vs Ag/AgCl reference electrode

The signal reproducibility and operational stability are the key characteristics of any biosensor. To determine reproducibility of signals of the GlOx/silicalite biosensor, the responses to glutamate of two concentrations (0.5 and 1 mM) were measured over one working day with 20-min intervals; between measurements, the biosensor was kept in the buffer with continuous stirring (Fig. 9a). Relative standard deviations were 5.7 and 6.9%, respectively.

To study the operational stability of the biosensor, nine responses to 1 mM glutamate were measured step-by-step daily during 4 days. All the time between measurements, the biosensor remained in the buffer at continuous stirring; after a series of nine measurements, the biosensor was dried and placed in a refrigerator (+4 °C). As seen in Fig. 9b, the biosensor was characterized by good operational stability over 4 days.

## Conclusions

A new amperometric glutamate-sensitive biosensor has been developed on the basis of GlOx adsorption on the amperometric disk platinum electrode coated with a layer of silicalite. The optimal procedures of deposition of a silicalite layer on platinum electrode and GlOx adsorption on silicalite have been elaborated. It has been shown that the biosensor created in compliance with optimized conditions of immobilization has high sensitivity to glutamate (the minimum detection limit—0.5–1 μM, wide linear range of operation (2.5–400 μM) and is characterized by good reproducibility (error did not exceed 7%) and operational stability during 4 days. Summarizing all the results obtained, the conclusion can be made that the developed amperometric biosensor based on GlOx adsorbed on silicalite is a promising device for further successful application for glutamate analysis in real biological fluids.
